# Population Genetic Structure of a Widespread Bat-Pollinated Columnar Cactus

**DOI:** 10.1371/journal.pone.0152329

**Published:** 2016-03-25

**Authors:** Enriquena Bustamante, Alberto Búrquez, Enrique Scheinvar, Luis Enrique Eguiarte

**Affiliations:** 1 Departamento de Ecología de la Biodiversidad, Instituto de Ecología, Universidad Nacional Autónoma de México, Hermosillo, Sonora, Mexico; 2 Departamento de Ecología Evolutiva, Instituto de Ecología, Universidad Nacional Autónoma de México, México, D. F., Mexico; University of Arkansas, UNITED STATES

## Abstract

Bats are the main pollinators and seed dispersers of *Stenocereus thurberi*, a xenogamous columnar cactus of northwestern Mexico and a good model to illustrate spatial dynamics of gene flow in long-lived species. Previous studies in this cactus showed differences among populations in the type and abundance of pollinators, and in the timing of flowering and fruiting. In this study we analyzed genetic variability and population differentiation among populations. We used three primers of ISSR to analyze within and among populations genetic variation from eight widely separated populations of *S*. *thurberi* in Sonora, Mexico. Sixty-six out of 99 of the ISSR bands (*P* = 66.7%) were polymorphic. Total heterozygosity for all populations sampled revealed high genetic diversity (*H*_*sp*_ = 0.207, *H*_*BT*_ = 0.224). The AMOVA showed that most of the genetic variation was within populations (80.5%). At the species level, estimates of population differentiation, θ = 0.175 and θ^B^ = 0.194, indicated moderate gene flow among populations. The absence of a significant correlation between genetic and geographic distances indicated little isolation by geographic distance. The large genetic variation and diversity found in *S*. *thurberi* is consistent with its open reproductive system and the high mobility of bats, a major pollinator. However, small changes in number or kind of pollinators and seed dispersal agents, in the directionality of migratory routes, and/or in the timing of flowering and fruiting among populations, can critically affect gene flow dynamics.

## Introduction

Genetic diversity and its spatial distribution is the result of mutation, genetic drift, gene flow and natural selection acting within and among populations of a species [1, 2]. The relevance of these factors in determining genetic diversity is related to the heterogeneity of the physical environment, the intrinsic biological traits of the species (phylogenetic constraints), and the network of ecological interactions within the populations studied. Features such as the mode of reproduction and dispersal, and longevity have been related to variation and genetic structure in several groups of plants [3, 4]. For example, long-lived woody species show more genetic variation and a greater chance of spreading their genes both in time and space than herbaceous species [5] The reproductive systems of plants may also have a high impact on the genetic variability of plant populations. It has been demonstrated that cross-pollinated species usually have high diversity within populations and low population differentiation, whereas self-pollinated species show the opposite trend [3,5,6]. Furthermore, when pollen and seeds are transported over long distances, high levels of gene flow are expected [7]. Also, demographic factors influence the genetic structure of populations by reducing gene flow when populations are large [8,9].

In recent years, there has been a growing interest in the ecology, evolution and conservation of columnar cacti, many of which are not only an essential element of the ecological functioning of arid and semiarid communities of the New World, but are also fundamental elements of cultural and economic value for the people who inhabit these regions. Although the Cactaceae comprise nearly 1500 species and a high proportion of them are threatened with extinction [10], genetic variation of about only 27 species, mostly columnar cacti (23), has been studied [11–24]. Columnar cacti have high levels of genetic diversity and polymorphism, comparable to levels reported for long-lived woody species [5]. Genetic structure of columnar cactus populations correlates well with pollinating and dispersal agents [13]. For example, the high genetic differentiation among populations of *Lophocereus schottii* (Engelm.) Hunt, a columnar cactus of the Sonoran Desert, has been attributed to the limited dispersal abilities of its pollinator moth *Upiga virescens* [12], and to the restricted movements of the birds that disperse their seeds. In contrast, other columnar cactus species that depend for pollination and seed dispersal on bats usually show low differentiation among populations [13,16].

In plants that are pollinated by animals, foraging patterns have a major influence on patterns of gene flow [25,26]. These in turn are affected by the density of blooming plants and flowering phenology [27]. Empirical evidence suggests that pollen-mediated gene flow is the predominant form of gene flow in plants [28–30]. However, contributions of pollen and seeds to overall gene flow can vary on different spatial scales, and in some cases can contribute equally to gene flow [31]. Several species of columnar cacti are partially or completely dependent on bats and birds for pollination and seed dispersal. In these cases, gene flow seems to be highly dependent on both processes [32] through pollen carry-over in between successive flower visits, and by endozoocory as New World bats can travel long distances while seeds pass through their digestive systems, in some cases longer than two hours [33–35]. The uncertainty in the magnitude of gene flow due to pollen or seeds remains little studied and the effects of pollen and seed vectors are likely to change the pollen/seed migration ratios.

Organ pipe cactus, *Stenocereus thurberi* (Engelm.) Buxb., is a chiropterophilous columnar cactus, pollinated mainly by the bat *Leptonycteris yerbabuenae*, as well as by some species of birds and hawkmoths [36,37]. It is self-incompatible and shows geographic differences in reproductive success caused mainly by differences in the kind, abundance, and efficiency of pollinators [37]. As the food preferences of nectar-feeding bats shift towards frugivory later in the season, seed dispersal is also strongly dependent on bats [38]. Differences in the directionality of migration routes, the mobility of local pollinators, and different pollinator types can affect gene flow between populations of *S*. *thurberi* [37]. It is our basic hypothesis that since bats and some birds are highly vagile and can carry pollen and seeds far from maternal plants, we expect high genetic diversity within populations and low differentiation among continental populations assuming no major geographical barriers. We selected *S*. *thurberi* for the exploration of these hypotheses because of its widespread distribution, extending from the most xerophytic environments in the desert to mesic tropical dry forests, high local densities of plants across its distribution range, documented spatial variability in phenology and flower visitation, and the accessibility of its flowers and fruits for experimentation. We used the molecular marker ISSRs (Inter Simple Sequence Repeats) [39] to examine and compare levels and patterns of genetic variation along its continental range, and determine its genetic structure.

Like other columnar cacti, this species has been used by humans mainly for food, for medicinal purposes, and as a source of building material and fuel [40–42]. In addition, its fruits are sold in local and regional markets. Given its value as a keystone species and its economic potential, analyzing levels of genetic variability and its distribution among populations is important not only for exploring the evolutionary differentiation of a major species of this desert systems, but also to indirectly infer the effect of spatial mobility of its migrant and local pollinators and dispersers. This study is also important because of ongoing habitat fragmentation and destruction throughout its species range [43] that are likely affecting its ecologic and genetic structure, as well as the long term persistence of this iconic member of the Sonoran Desert and adjacent arid lands.

## Materials and Methods

### Study species

Organ pipe cactus, *S*. *thurberi*, also called pitaya dulce, is a columnar cactus 3 to 8 m high, with numerous vertical stems (arms) that emerge from the base or from a small trunk [44]. It is diploid (2n = 22) [45] and occurs from northern Sinaloa and western Chihuahua to southwestern Arizona. It is also found across most of the southern half of the Baja California peninsula [44,46]. Southern populations are large and cover many square kilometres, but in its northern and western limits, populations are smaller and individuals tend to be more scattered [44]. The creamy white to pink flowers are perfect, solitary, and with a morphology suggesting bat-pollination. The flowers open at night and close during the following morning. Flowering starts in mid-May, although sometimes begins in late April, and lasts for 10 weeks or more [46]. Flowering coincides with the arrival of migratory bat *L*. *yerbabuenae* that concentrates during winter in southern and central Mexico [47]. Bats are the main pollinators of *S*. *thurberi*, but several species of birds and hawkmoths also play an important role in pollination [37]. The fruits ripen throughout the summer and offer a tasty reward to dispersal agents, usually bats and birds [38].

### Study Area

**Fig 1:**
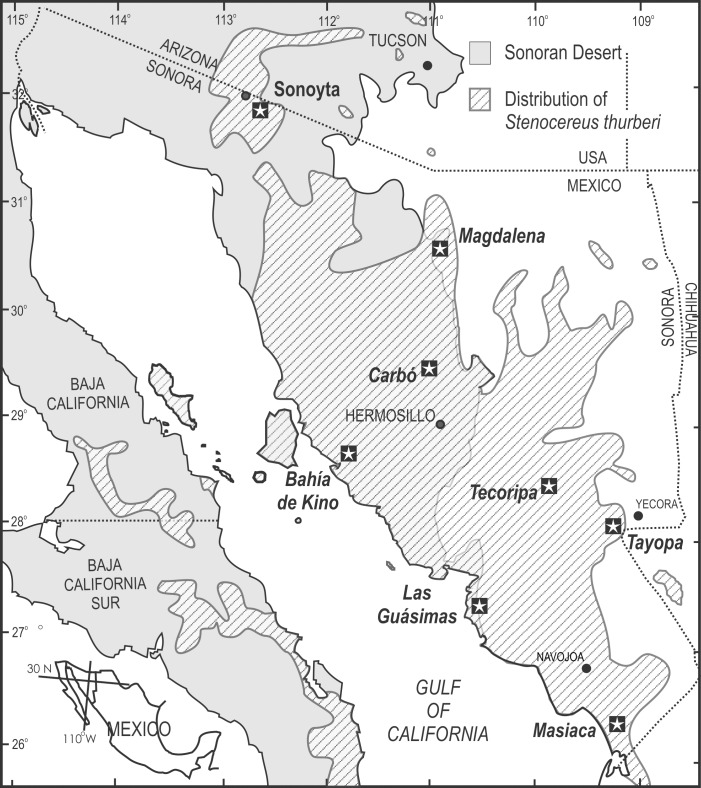
Geographical distribution of *Stenocereus thurberi* (diagonal lines; modified from Turner *et al*., 1995), and location of the eight studied populations (stars). Sonoran Desert extent (gray).

We sampled 8 populations covering the entire range of the continental populations of *S*. *thurberi* (Fig 1). These populations are located from sea level to nearly 1000 m elevation, and experience from about 100 to over 400 mm rainfall. Some populations are exposed to occasional freezing temperatures and range from the hyper-arid desert to the tropical deciduous forests of the foothills of the Sierra Madre Occidental ([48]; Table 1).

**Table 1:** Geographic location, elevation and vegetation type of *Stenocereus thurberi* populations used for genetic analysis in this study.

Population	*N*	Latitude N	Longitude W	Elevation (m)	Vegetation
Sonoyta	44	31° 48´ 17´´	112° 51´ 56´´	512	Arizona Upland^1^
Magdalena	45	30° 40´ 57´´	110° 58´ 46´´	964	Arizona Upland^1^
Carbó	42	29° 34´ 06´´	111° 05´ 29´´	501	Plains of Sonora^1^
Bahía de Kino	30	28° 52´ 56´´	112° 01´ 39´´	60	Central Gulf Coast^1^
Tecoripa	30	28° 37´ 50´´	109° 57´ 42´´	411	Foothills Thornscrub^3^
Las Guásimas	43	27° 52´ 10´´	110° 28´ 55´´	5	Central Gulf Coast^1^^,^^2^
Tayopa	40	28° 20´ 16´´	109° 13´ 06´´	730	Tropical deciduous forest^3^
Masiaca	43	26° 38´ 12´´	109° 18´ 39´´	15	Coastal scrub^3^

*N* = number of sampled individuals.

^1^Sonoran Desert according to Shreve [49].

^2^Transition with coastal scrub.

^3^Not part of the Sonoran Desert [48].

### Ethics Statement

The work met the Mexican legal requirements and was approved by Dirección General de Gestión Forestal y de Suelos, Secretaría de Medio Ambiente y Recursos Naturales (SEMARNAT) under collection permit SGPA/DGGFS/712/3133/13. Tissue samples were obtained as to minimize plant damage, allowing for rapid healing. In all cases, we obtained the permission of land owners to sample within their property.

### Population sampling

We collected a narrow strip of photosynthetic tissue of approximately 10 cm long and 1–2 cm wide from the ribs of young stems from about 40 reproductive individuals in each of the selected populations. Individual plants were chosen arbitrarily along two orthogonal axes as they appeared in the field. To avoid the rare and unlikely case of vegetative propagation, when two individuals were less than two meters apart, we only collected one of these. Tissue samples were stored in the field in an ice chest. Later that day, these were transferred to a freezer at -19°C before storing in an ultracold freezer (-80°C).

### DNA extraction

DNA extraction was accomplished using mini-preps [50] with some modifications for succulents [51]. About 1 g of tissue was ground with liquid nitrogen to a fine powder. Genomic DNA was extracted adding 900 μl of ethanol, 200 μl of CTAB extraction buffer, 750 μl of buffer STE, and 65 μl of 20% SDS. The mixture was then stirred and subsequently centrifuged at 12,000 rpm for 8 minutes. The supernatant was eliminated and the pellet resuspended in 250 μl of 2X CTAB buffer and 750 μl of STE buffer. The samples were centrifuged again at 12,000 rpm for 8 minutes, the supernatant was eliminated and the pellet resuspended in 400 μl of 2X CTAB buffer and 600 μl of STE buffer. Samples were centrifuged again at 10,000 rpm for 8 minutes, the supernatant eliminated, and the pellet was resuspended with 600 μl of 2X CTAB buffer. The samples were treated with 7000 u /ml (25 μl per sample) of ribonuclease at 37°C for 20 minutes to digest the RNA. After this treatment, 50 μl of proteinase-K (20 mg/mL) was added and the sample was incubated at 65°C for 30 minutes. Samples were placed on ice for 15 minutes to stop the reaction. DNA was isolated by adding 600 μl of chloroform:octanol (24:1) per sample, homogenized and centrifuged at 9000 rpm for 12 minutes, and the supernatant was transferred to a new centrifuge tube. DNA was precipitated with 600 μl of cold isopropanol (-20°C) and maintained for 12 hours at -20°C. After this time, the samples were centrifuged at 12,500 rpm for 7 minutes and the supernatant was discarded. The pellet was washed by adding 1 ml of cold ethanol (-20°C) to 70% and centrifuged at 14,500 rpm for 5 minutes. The supernatant was removed and the dried pellet was resuspended in 100 μl of ultrapure water for PCR and stored at 4°C. We quantified the DNA concentration of the samples using a BioPhotometer (HQ Eppendorf, Hamburg, Germany).

### Inter-simple sequence repeats (ISSR) amplification

A total of 20 random ISSR primers (Series No. 9, University of British Columbia) were screened. The random amplified ISSR best products were tested again by adjusting PCR conditions including amount and concentration of Mg, dNTP's, primer and DNA, as well as the times and temperatures of alignment and extension. Three ISSR random primers were selected to search for genetic variation that were present in all studied populations, were not monomorphic, and showed a clear and reproducible pattern of bands (loci): 835 [(AG) _8_YC, 846 [(CA) _8_RT] and 850 [(GT) _8_YC] where R is equivalent to Adenine (A) or Guanine (G) and Y to Cytosine (C) or Thymine (T).

PCR conditions for primer reactions in a total volume of 25 μl consisted of 80ng/μl of DNA (4 μl), IX PCR buffer (2.5 μl), 0.2 mM of each dNTP (2 μl), 0.5 μM of primer (1.25 μl), 1 unit of Taq polymerase (0.2 μl), 2.25 mM of MgCl_2_ for primer 835 (1.87 μl), 1.25 mM of MgCl_2_ for primer 846 (1.4 μl) and 1.75 mM of MgCl_2_ for primer 850 (1.46 ul), and ultrapure water for PCR.

All reactions were conducted in a Thermo Hybaid PCR Express thermocycler with the following amplification program: initial denaturation was carried out for 4 minutes at 95°C, followed by 36 cycles of 40 seconds at 94°C, 45 seconds at a temperature of optimal alignment (55.5°C for primer 835, 52.5°C for primer 846 and 51°C for primer 850), 75 seconds of extension at 72°C, and finally 5 min at 72°C, followed by a period standing at 4°C. We included a negative control tube in each reaction to verify that the reagents used were free of DNA contamination. As a positive control, we compared the resulting PCR reactions with repetitions for at least two individuals of each population and primer to confirm data repeatability. The amplification products were stored at 4°C until they were electrophoresed in 180 ml of 2% agarose suspended in 1X TAE buffer (Tris-Acetic acid-EDTA) with a constant voltage of 180 V. The gels were visualized using an ultraviolet lamp after staining with 1.0 μg/ml ethidium bromide and photographed with a Kodak EDAS 240 digital camera. Images were processed using the Kodak 1D Image Analysis software version 3.5 (Scientific Image Systems, Eastman Kodak Company). The size of the fragments was determined by comparison with a molecular weight marker (Ladder) of 100 base pairs (Invitrogen mark) of 250μg (1.0 μg/μL). The patterns of the amplified loci were read directly from the gels. Only the bands that showed consistency with the amplification were considered; blurred and weak bands were ignored. We constructed a data matrix of individual presence/absence of bands for each primer in each population, with each band presence/absence considered a genotype. Statistical analyses were performed using these matrices (see S1 Dataset). Individuals who did not amplify for one or more primers were not included in the analysis.

### Genetic Variation

Dominant markers such as ISSRs do not distinguish between homozygous and heterozygous genotypes, therefore the estimate of allele frequencies directly from the gel was not possible. To reduce bias in the estimation of allele frequencies, Lynch and Milligan’s [52] corrections for dominant markers were applied to exclude the bands whose frequency exceeded *1-(3/N)*, where *N* is the number of individuals sampled. This was done to avoid selecting loci with high frequencies of null alleles. Assuming that the population is in Hardy-Weinberg equilibrium (HWE), the frequency of null allele (*q*, recessive) was calculated as the square root of the frequency of the homozygous recessive (*q = x*^*½*^), which is represented as the absence of band (*x*), and the frequency of marked allele (*p*, dominant) is equal to *1-q*. Genetic variation of the populations was estimated using the heterozygosity and the proportion of polymorphic loci. Using the correction for dominant markers proposed by Lynch and Milligan [52], the expected heterozygosity for each locus was obtained from the formula: *H*_*e*_ *= 2q(1-q) + 2Var(q)*, where *Var(q) = (1-x)/4N* and the allele frequency of *q* is calculated from the formula: *q = x*^*½*^*[1-Var (x)/8x*^*2*^*]*^*-1*^ and *Var(x) = x(1-x)/N*, where *N* is the number of individuals in the sample [53]. Considering each of the bands as a locus, we calculated the proportion of polymorphic loci (*P*) as the frequency of the more frequent allele in the population ≥ 0.95. The polymorphism of a population was estimated as the total number of polymorphic loci divided by the total number of loci studied. Polymorphism varied from 0 (none of the loci is polymorphic) to 1 (all loci studied are polymorphic).

To determine whether there were significant differences in allele frequencies observed between populations, the exact test of population differentiation [54] was applied as implemented in TFPGA version 1.3 [55] using 1000 dememorizations, 10 batches and 2000 permutations per batch. In addition, a Mantel analysis [56] was used to estimate the correlation between genetic and geographic distances of populations.

### Genetic structure and gene flow

Assuming HWE, we calculated the coancestry parameter theta (*θ*) with TFPGA 1.3 [55] that uses the algorithm of Weir and Cockerham [57], equivalent to Wright’s *F*_*ST*_, a measure of the degree of genetic differentiation among populations. To estimate confidence interval of 95% a jackknife and a bootstrap with 5000 replications was performed. To calculate the variance within and among populations, we used AMOVA using Arlequin ver. 3.1 [58] following Crow and Aoki [59]; this model does not use allele frequencies or assume HWE. This analysis provided estimates of the proportion and significance of the variability explained by each hierarchical level allowing the calculation of *Ф*_*ST*_, an analogue of *F*_*ST*_. The variance components were tested for statistical significance by nonparametric randomization tests using 1023 permutations.

We also calculated genetic diversity and its distribution within and among populations with Bayesian methods for dominant markers that do not assume that genotypes within populations are in HWE [60,61]. The Bayesian estimator *H*_B_ of genetic diversity, comparable to Nei’s [62] genetic diversity or expected panmictic heterozygosity (*Hs*) and the Bayesian estimator of population structure θ^B^, directly comparable to estimates of the Wright’s [63] *F*_ST_ based on the θ of Weir and Cockerham [57], were calculated using the Hickory program ver. 1.1 [64]. We used the *f-free* model (suggested for dominants markers) that chooses values of *f* (inbreeding coefficient) at random, incorporating all of the uncertainty in the prior of *f* in the parameters obtained. We used the recommended default settings [64]: burn-in = 5000, number of samples = 25,000 and thinning factor = 5. For each population, we performed the Gelman-Rubins convergence test of Markov Chain Monte Carlo (MCMC) with the hdrcde package for R (Highest density regions and conditional density estimation; Rob J. Hyndman, Jochen Einbeck and Matt Wand). The converged chains were concatenated and density curves were estimated to obtain the modal values of θ^B^ for each population at 95% credibility intervals (CrI, equivalent to confidence intervals).

Population structure for the total data set was further investigated with a Bayesian clustering analysis conducted in STRUCTURE ver. 2.3.2 [65,66]. In this analysis, individuals were assigned probabilistically to one of the predefined *K* populations (gene pools) to identify the optimal number of genetic groups [67]. The optimum number of groups (*K*) was determined by varying the value of *K* from 1 to 12 and running the analysis 25 times per *K* value, in order to determine the maximum value of posterior likelihood [lnP(D)]. Each run was performed using 25000 burn-in periods and 25000 MCMC repetitions after burn-in. We used a model allowing admixture with correlated allele frequencies without any prior information. We determined the most probable *K* value using the maximum value of ΔK according to Evanno *et al*. [67].

### Genetic distance and isolation by distance

To assess genetic similarities between all pairs of populations, we estimated the linear function Fst/(1-Fst) [68] using the R library gstudio [69]. Isolation by distance was tested by the regression using a Mantel test with 10,000 permutations of the pairwise estimates of the linear function Fst/(1-Fst) against the log-transformed geographic distance using the Vegan R library [70]. The similarities between populations were set by the unweighted pair group method with the arithmetic mean (UPGMA), validating the branches with 1000 bootstrap permutations.

## Results

### Genetic variation

From the amplifications generated with the three ISSR primers, we obtained 99 bands (“loci”): 36 with primer 835, 27 with primer 846 and 36 with primer 850. We found significant differences in allelic frequencies among populations for 88 of 99 loci examined (exact differentiation test, *P* < 0.05).

**Fig 2:**
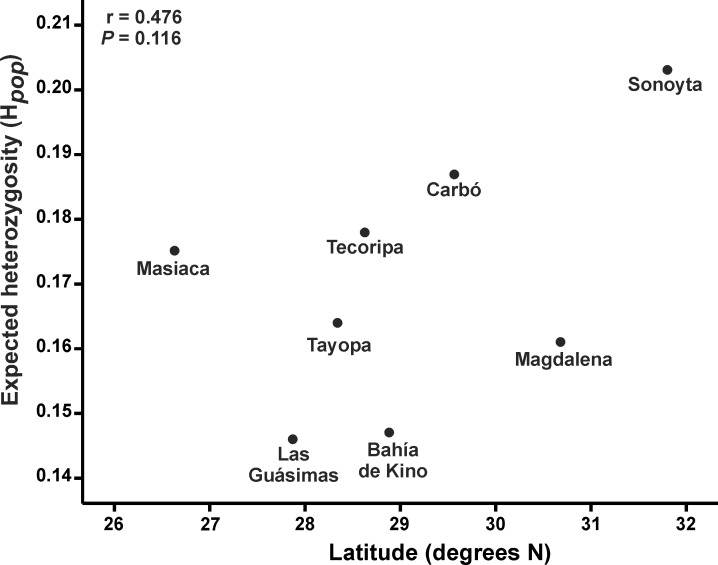
Relationship between average population heterozygosity (*H*_*pop*_) and latitude for eight populations of *Stenocereus thurberi*.

On average, we found that for the total of the populations studied, *S*. *thurberi* displayed relatively high percentages of genetic variation, both measured as expected heterozygosity and polymorphic loci *(H*_*sp*_ = 0.207±0.016 S.E., *P* = 66.66%, *H*_*BT*_ = *0*.*224*±0.009 S.D.). At the population level, the northern population of Sonoyta showed the maximum value of genetic variation (*H*_*pop*_ = 0.203±0.018 SE, *P* = 61.62%), while the minimum was found in the southern population of Las Guásimas (*H*_*pop*_ = 0.146±0.018 SE, *P* = 41.41%; Table 2). No significant differences were found when comparing levels of expected heterozygosity among populations (ANOVA: *F*_*7*,*784*_ = 1.261; *P* = 0.267), nor for any correlation between values of expected heterozygosity of each population with its latitude, longitude or altitude (*P*>0.1). The highest correlation (although statistically not significant) was found between expected heterozygosity and latitude (*r*^*2*^ = 0.227, *P* = 0.116), with a general trend to increased heterozygosity at higher latitudes (Fig 2). The Bayesian genetic diversity estimator *H*_*B*_ shows the same results, the highest values in the Sonoyta population (0.214±0.008 SD), and the lowest in the Las Guásimas population (0.160±0.006 SD; Table 2).

**Table 2:** Total and within population genetic variation in *Stenocereus thurberi*.

Population	*N*	*H*_pop_	%*P*	*H*_*B*_	*H*_*B*_, CrI 95%
Sonoyta	44	0.203±0.018	61.62	0.214±0.008	0.200–0.230
Magdalena	45	0.161±0.017	53.53	0.169±0.006	0.157–0.183
Carbó	42	0.187±0.017	56.56	0.208±0.012	0.188–0.231
Bahía de Kino	30	0.147±0.018	44.44	0.171±0.007	0.158–0.184
Tecoripa	30	0.178±0.018	54.54	0.196±0.007	0.182–0.210
Las Guásimas	43	0.146±0.018	41.41	0.160±0.006	0.148–0.173
Tayopa	40	0.164±0.016	54.54	0.187±0.010	0.169–0.208
Masiaca	43	0.175±0.017	53.53	0.191±0.009	0.175–0.211
*Total*	317	*H*_*sp*_ = 0.207±0.016	66.66	*H*_*BT*_ = 0.224±0.009	0.210–0.243

*N* = number of sampled individuals: *H*_pop_ (± SEM) = expected heterozygosity assuming Hardy-Weinberg equilibrium (HWE); *H*_*sp*_ = diversity for the total species; %*P* = percentage of polymorphic loci assuming HWE with the 95% criterion; *H*_*B*_ (± SD) = Bayesian expected panmictic heterozygosity not assuming HWE; *H*_*BT*_ = diversity in the entire pool; *CrI* = credibility interval.

### Genetic structure and gene flow

Variation among populations was compared using the coancestry coefficient (*θ*) that ranged from *θ* = 0.137–0.212 with a confidence level of 95%. The average value of *θ* for all loci was 0.175, indicating moderate genetic differentiation. The AMOVA revealed that most of the genetic variation was found within populations (80.49%), resulting in a value of *Ф*_*ST*_ = 0.195, analogous to *F*_*ST*_ (Table 3). The values of *Ф*_*ST*_ and θ are similar to the Bayesian genetic structure value (θ^B^ = 0.194) and are within the 95% CrI (0.163–0.226, Table 3).

**Table 3:** Summary of analysis of molecular variance (AMOVA) performed with 99 loci ISSRs in eight populations of *Stenocereus thurberi* and genetic differentiation estimators analogous to Wright’s [63] F statistics.

	AMOVA [71]					*F*_*ST*_ Bayesian estimator [57]			*F*_*ST*_ estimator [54]		
Source of variation	d. f.	SS	VC	%V	*Ф*_ST_	θ^B^	SD	CrI	θ	SD	CI
Among populations	7	728.323	2.384	19.51*	0.195	0.194	0.0160	0.163–0.226	0.175	0.019	0.137–0.212
Within populations	309	3040.63	9.840	80.49*							
*Total*	*316*	*3768*.*953*	*12*.*224*								

SS = sum of squares, VC = variance component, %V = percentage of variation; SD = standard deviation; CrI = credibility interval; CI = confidence interval.

* P<0.001.

**Fig 3:**
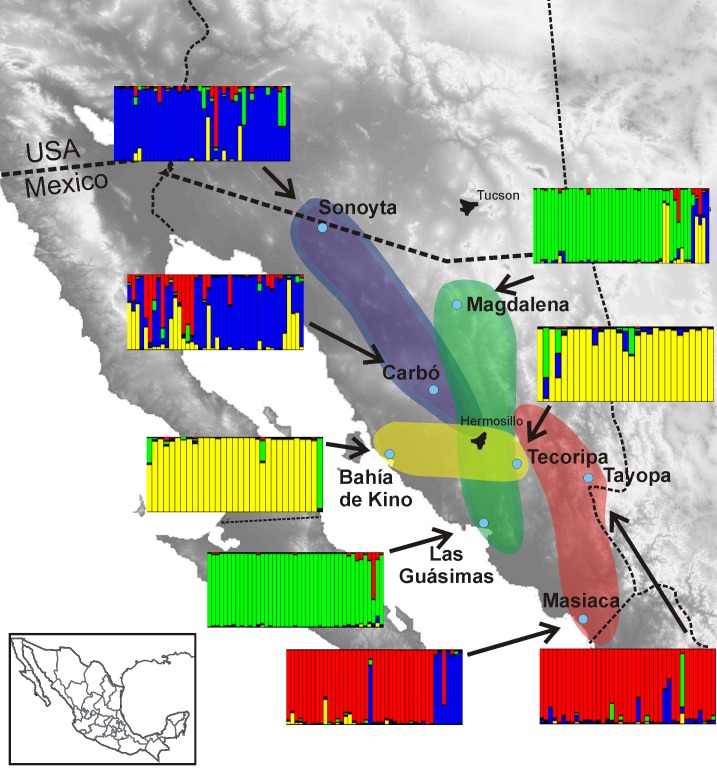
Resultant groups from STRUCTURE. Note the multiple SE-NW grouping, and the marked E-W group in central Sonora.

The most probable number of groups (K) derived from STRUCTURE analysis was four, however, other groupings were less likely. The four groups suggested: 1) three groups with a predominantly SE-NW direction including in the southeast, Masiaca-Tayopa; in central Sonora, Las Guásimas-Magdalena; and in the northwest, Carbó-Sonoyta, and 2) one group in central Sonora linking the Sierra Madre foothills and the coast of the Gulf of California, Tecoripa-Bahía de Kino, overlapping the other three groups with a predominant E-W trend (Fig 3). A hierarchical AMOVA including the four groups defined from STRUCTURE ascribed about 79.4% of the genetic variation within populations, and significantly partitioned the remaining variance of 9.0% among geographical groups, and 11.6% among populations within groups (S2 Table).

### Genetic distance between populations

**Fig 4:**
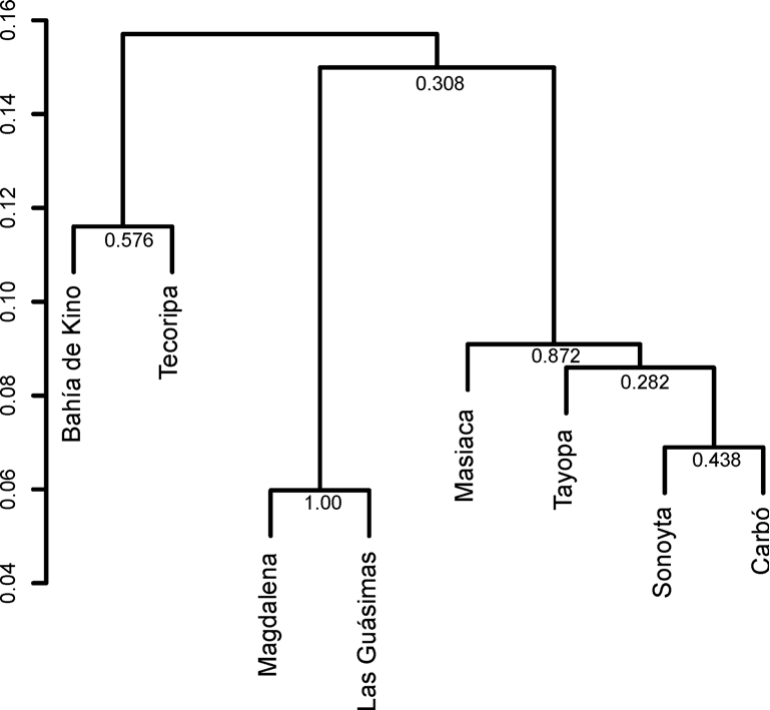
Dendrogram (UPGMA) showing the relations among the studied populations of *S*. *thurberi* using the Rousset’s [68] genetic distance Fst/(1-Fst). At each node is the value of bootstrap that supports it.

Genetic distances expressed as the linear regression Fst/(1-Fst) [68] between populations were small. They ranged from 0.060 to 0.208, where Magdalena and Las Guásimas were the closest genetically, and the two most distant populations, Sonoyta and Kino, were the most genetically unrelated populations (S1 Table). The average genetic distance between all pairs of populations was 0.134. The UPGMA produced the same pattern as STRUCTURE (Fig 4). The grouping of populations created by a neighbor-joining analysis was similar to that found by the UPGMA (S1 Fig). Thus, these populations of *Stenocereus thurberi* did not follow a pattern of isolation by distance (Mantel test, r = -0.214; *P* = 0.824).

## Discussion

### Genetic diversity

Columnar cacti are characterized by relatively high levels of genetic variability, and *S*. *thurberi* is not an exception (*P*_sp_ = 66.7%, *H*_sp_ = 0.207). Our results are consistent with those of Hamrick *et al*. [13] using isozymes. However, our data provides an explicit spatial context to differences in population structure, while Hamrick *et al*. [13] assessed mean values for mainland vs peninsular populations. Our estimated values of genetic variation, both at the species and population level, were within the moderate to high range of genetic variability observed in the few other studies of columnar cacti (S3 Table). As these results were obtained with a host of different genetic markers, any comparisons should be taken with caution. These comparisons comprise species with similar life histories and ecological traits as *S*. *thurberi*, i.e. all are long-lived woody species with wide geographical distributions, high population densities, predominantly outcrossing mating systems, and animal dispersal of pollen and seeds over long distances. All these features have been associated with high levels of genetic diversity of vascular plants in general [3,72,73] and of columnar cacti in particular [16,17,22].

In *S*. *thurberi* populations, higher genetic variation in northern populations was surprising as these populations were more recently established (between 5.2 and 3.5 ka B. P.) than more southerly populations [74]. A possible explanation for these high levels of genetic variation in the north is that these populations were established from migrants derived from different southern refugia in central Sonora.

### Genetic structure and gene flow

An open pollination system and long-distance seed dispersal can substantially promote gene flow among populations. In the case of *S*. *thurberi*, bats, birds and insects have been associated with the movement of pollen and/or seeds [36–38], most likely reducing population structuring and retaining high levels of genetic diversity. However, *S*. *thurberi* populations show relatively higher genetic differentiation than most species of chiropterophilous cacti (S2 Table). Our results are comparable with cactus species pollinated by insects (*Pereskia guamacho*, *G*_ST_ = 0.112;[75]), and hummingbirds (*Melocactus curvispinus*, *G*_ST_ = 0.189; [32]), and are almost three times higher than the previously reported mean value for several continental populations of *S*. *thurberi* [13]. Their extensive species range, complex phenological differentiation among populations [46], and the strong spatial and temporal variation of different groups of pollinators [37] may explain some incipient genetic differentiation. Such is the case of southern populations with a much longer flowering phenology [46], and the greatest temporal and spatial variation in the types of pollinators [37]. In contrast, in the northern populations, flowering is short and the only observed pollinators were bats. Thus, bats, which are highly mobile and travel long distances (pollinating flowers, and later dispersing seeds), seem to promote lower levels of genetic differentiation (i. e. homogenize the populations through gene flow [16]). Hawkmoths, perching birds, and hummingbirds can exert an important force in reproductive success, and could lead to greater genetic differentiation because of their shorter flying bouts, territorial habits, and less specific resting places that tend to maintain gene flow within the local population neighbourhood.

Moderate values of gene flow can be explained in part because the long nosed bat, *L*. *yerbabuenae*, is one of the main pollinators of *S*. *thurberi* [36,37]. This bat species has high mobility and is capable of flying long distances to forage, up to 100 km in one night in the Sonoran Desert [76]. It also maintains certain migration routes, probably dictated by the location of caves where females give birth and nurse their offspring [76,77], and by the heterogeneous distribution of sheltering sites in the desert. Seed dispersal, mostly by bats, but also by perching birds like Gila woodpeckers (*Melanerpes uropygialis*) and house finches (*Carpodacus mexicanus*), is likely to represent a significant, albeit small, fraction of gene flow. However, other organisms are known to be involved in seed dispersal. For example, it has been documented that humans are likely responsible for long-distance dispersal of *Pachycereus pringlei*, and probably for other columnar cacti [78]. Indigenous peoples of northwestern Mexico and southwestern USA with an ancient tradition of consumption of fruits of columnar cacti, undoubtedly have had an impact on the distribution, abundance, and gene flow of *S*. *thurberi* through gene flow by seed dispersal. As in the case of bats, certain routes determined by water, food, and shelter availability could provide corridors for preferential gene flow that might obscure isolation-by-distance patterns [78,79]. Bats, birds and humans are efficient dispersers, but differ in their ability to move pollen and/or seeds among the populations studied. Average genetic differentiation among populations is only a reflection of the importance of the different vectors of pollen and seeds. In populations primarily pollinated and dispersed by bats we might expect little differentiation, whereas in populations characterized by a shortage of pollinators and dispersal agents, greater differentiation and probably less genetic variation might occur.

In the absence of isolation by distance [80], moderate gene flow between populations could be related to ecological factors, such as changes in the timing of flowering and fruiting among populations [46]. Given that many Sonoran Desert plants have recently moved from southern refuges after the last glaciation [14,81], it is likely that the time after migration plays a role in the modest differentiation among populations, particularly in the northern range of distribution. Consequently, there might have been insufficient time to achieve equilibrium in some populations (80 to 160 generations since last glacial period). However, the extensive movement of bats is probably an important factor affecting the genetic makeup of this species.

As Cockrum [82] demonstrated, migratory bats do not follow a simple route from south to north in northwestern Mexico, but perform complex movements, pursuing flowering of the Sierra Madre Occidental agaves and columnar cacti in the desert. In their migration north, bats also pollinate some trees of the northern dry deciduous forests of Sonora such as *Ceiba aesculifolia* and *Bombax palmeri*. The complexity of this pattern was confirmed by Bustamante *et al*. [37], who showed that northern populations are visited only by bats. In these populations, bats are always present throughout the flowering season, while in the southern populations, birds and hawkmoths are the sole visitors at the beginning of flowering, and bats only appear at the end of the reproductive season. Therefore, it is likely that, due to the complex migratory routes of bats, the patterns of gene flow and differentiation of *S*. *thurberi* would be far more intricate than simple geographical distance. The results from STRUCTURE suggest there are links among populations along a S-N axis, as well as an E-W axis, suggesting 1) complex patterns of gene flow, most likely related to bat local and global migration, and 2) different groups of pollen and seed vectors that could overlap in time and space. In the first case, local and regional migration patterns depend on the availability of resources and adequate sites for giving birth and tending the young bats [82]. The results of Ramírez [83] that showed northwest-southeast corridors, and an east-west corridor of migrating bats in central Sonora are strikingly coincident with our findings, and lend support to the notion that gene flow occurs in several directions at different times. In a regional sense, bats migrate from southeast to northwest following the progression of the flowering season. For example, the first species of columnar cacti flowering along the base of the Sierra Madre Occidental at the end of the winter season in northern Sinaloa is *Pachycereus pecten-aboriginum*. During spring, some chiropterophilous species (*Stenocereus montanus*, *Ceiba aesculifolia* and *Bombax palmeri*, among others) bloom in the foothills of southern Sonora. Then, near the onset of summer, the northern species of columnar cacti initiate flowering in the desert plains. At a local level, migration becomes diffuse and complex because several species of bat pollinated cacti flower almost synchronically: first *Pachycereus pringlei* (cardón-sagüeso) in coastal Sonora and the Baja California peninsula, and *Carnegiea gigantea* (saguaro) in Sonora and Arizona, and later *Stenocereus thurberi* throughout Sonora and Baja California Sur. Results from STRUCTURE analyses hinted that there could be several groups of bats following corridors northwards, or coming from the Sierra Madre Occidental outposts towards the rich coastal forests of cardón, saguaro, and organ pipe cactus. The patterns from STRUCTURE are strongly supported by the genetic distance analysis (UPGMA and neighbour-joining) that show high correspondence in population groups. The migrating bats most likely go across the Gulf of California via the midriff islands as shown by Ramírez [83] in her study of mitochondrial DNA of bats. The presence of corridors used by different waves of migrant bats greatly obscures the isolation by distance hypothesis. The picture is further muddled because bats (pollinators and seed dispersal agents of several columnar cacti of the Sonoran Desert) that head north pollinating flowers could also fly south to take advantage of mature fruits before flying north again when another species of columnar cacti reaches its flowering peak.

## Conclusions

Levels of genetic variability and genetic structure of *S*. *thurberi* are mainly the products of the activity of their pollinators and dispersers, as well as the complex history and particular ecological settings of each population. The life history traits of *S*. *thurberi* (obligately xenogamous, long-lived perennial with a large geographic range) can contribute to the maintenance of high levels of genetic diversity. These results support previous observations that columnar cacti have moderate levels of genetic variability.

The movement of pollinators and dispersal agents highlight the role of gene flow as an evolutionary force [84]. The lack of correlation between genetic and geographic distances of populations may be due to other ecological features, such as type of pollinator and seed dispersers, the variable flowering phenology, and the migratory routes of pollinators. Several migration corridors are apparent, as the bats that are the more important pollinator and seed dispersal agents of the species migrate from south to north following the progression of the flowering season and the flowering and fruiting of different species. However, at a local level, migration and gene flow among populations of organ pipe cactus becomes diffuse and complex, and our results may suggest the existence of several groups of bats following different corridors northwards, or flying back and forth from the Sierra Madre Occidental towards the coast, as Wilkinson and Fleming [85] first proposed for the coastal and inland corridors. More recently Ramirez [83] further refined the genetic structure of bat populations, and showed bats heading north while pollinating flowers are likely to fly south to consume mature fruits before flying back north, west, or east.

Levels of genetic diversity suggest that populations of *S*. *thurberi* are repositories of most of the species’ genetic variation. Therefore, we might be tempted to think that keeping a few populations should be sufficient to maintain the current genetic diversity. However, this approach does not consider the need to maintain pollination corridors that allow migration of bats, its main pollinators and dispersal agents. These corridors are complex and seem to depend on the local community dynamics. Even though *S*. *thurberi* has relatively high levels of genetic diversity, the persistence of this species depends on the activities of migratory organisms for their pollination and dispersal, and is threatened by deforestation and fragmentation of the landscape along its distribution range [43].

*Stenocereus thurberi* is present in both sides of the Gulf of California. In this study, we only examined the populations in the continental distribution range. Future research needs to include peninsular and island populations to better understand bat migration patterns, and to elucidate the phylogeographic structure and origin of these disjunct populations which offer the chance to explore the contrast between dispersal and vicariance associated with the formation of the Gulf of California.

## Supporting Information

S1 Dataset Matrix of presence/absence data for each population and all sampled individual using 99 loci. (XLS)

S1 Fig Dendrogram (BIONJ method, Gascuel, 1997) showing the relatedness among the studied populations of *S*. *thurberi* using the Rousset’s genetic distances Fst/(1-Fst). At each node is the value of bootstrap (based in 1000 replicates) that supports it.
(TIF)

S1 Table Rousset’s genetic distances Fst/(1-Fst) (above diagonal), and geographic distances in km (lower diagonal) among the eight populations studied of *Stenocereus thurberi*. (DOCX)

S2 Table Analysis of molecular variance (AMOVA) showing the partitioning of genetic variation within and among groups of *Stenocereus thurberi* defined for STRUCTURE. (DOCX)

S3 Table Estimates of genetic diversity at the species and population level in 23 species of columnar cacti reported until 2015. (DOCX)
