# Supplementary material for: Population Genetic Structure of a Widespread Bat-Pollinated Columnar Cactus
Source: PLoS One. 2016 Mar 25;11(3):e0152329. doi: 10.1371/journal.pone.0152329 (PMC4820105; doi:10.1371/journal.pone.0152329)
Supplement: S1 Table — (DOCX) [file pone.0152329.s003.docx]

**S1 Table.** Rousset’s genetic distances Fst/(1-Fst) (above diagonal), and geographic distances in km (lower diagonal) among the eight populations studied of *Stenocereus thurberi*.

|  | 1 | 2 | 3 | 4 | 5 | 6 | 7 | 8 |
| --- | --- | --- | --- | --- | --- | --- | --- | --- |
| 1 Sonoyta | - | 0.0977 | 0.0689 | 0.2082 | 0.1231 | 0.1422 | 0.0787 | 0.1113 |
| 2 Magdalena | 219 | - | 0.1069 | 0.1625 | 0.1188 | 0.0598 | 0.1463 | 0.1584 |
| 3 Carbó | 301 | 124 | - | 0.1545 | 0.1045 | 0.1308 | 0.0933 | 0.0721 |
| 4 Bahía de Kino | 335 | 224 | 119 | - | 0.1160 | 0.1824 | 0.1983 | 0.1776 |
| 5 Tecoripa | 450 | 249 | 152 | 203 | - | 0.1468 | 0.1642 | 0.1441 |
| 6 Las Guásimas | 495 | 317 | 198 | 188 | 99 | - | 0.1954 | 0.1917 |
| 7 Tayopa | 522 | 311 | 228 | 281 | 80 | 134 | - | 0.0894 |
| 8 Masiaca | 671 | 478 | 370 | 366 | 231 | 179 | 189 | - |
