# Supplementary material for: Population Genetic Structure of a Widespread Bat-Pollinated Columnar Cactus
Source: PLoS One. 2016 Mar 25;11(3):e0152329. doi: 10.1371/journal.pone.0152329 (PMC4820105; doi:10.1371/journal.pone.0152329)
Supplement: S2 Table — (DOCX) [file pone.0152329.s004.docx]

**S2 Table.** Analysis of molecular variance (AMOVA) showing the partitioning of genetic variation within and among groups of *Stenocereus thurberi* defined for STRUCTURE.

| **Source of variation** | **d. f.** | **SS** | **VC** | **%V** | ***P*** |
| --- | --- | --- | --- | --- | --- |
| Among groups | 3 | 461.871 | 1.113 | 8.99 | <0.01 |
| Among populations within groups | 7 | 266.452 | 1.434 | 11.57 | <0.001 |
| Within populations | 309 | 3040.63 | 9.840 | 79.44 | <0.001 |
| *Total* | *316* | *3768.953* | *12.387* |  |  |

d. f. = degree of freedom, SS = sum of squares, VC = variance component, %V = percentage of variation, *P*-value is based in 1023 permutations.
