# Supplementary material for: Population Genetic Structure of a Widespread Bat-Pollinated Columnar Cactus
Source: PLoS One. 2016 Mar 25;11(3):e0152329. doi: 10.1371/journal.pone.0152329 (PMC4820105; doi:10.1371/journal.pone.0152329)
Supplement: S3 Table — (DOCX) [file pone.0152329.s005.docx]

**S3 Table. Estimates of genetic diversity at the species and population level in 23 species of columnar cacti reported until 2015.**

| Species | Molecular marker | N | Genetic variation | | | | Locality | Source |
| --- | --- | --- | --- | --- | --- | --- | --- | --- |
|  |  |  | %*P*_sp_ | %*P*_pop_ | *H*_sp_ | *H*_pop_ |  |  |
| *Carnegiea gigantea* | Isozymes | 16 | 93.3 | 54 | 0.129 | 0.116 | Sonoran Desert Region | [1] |
| *Cereus repandus* | Isozymes | 14 | 94.1 | 71 | 0.242 | 0.170 | Northwestern Venezuela | [2] |
| *Escontria chiotilla* | Isozymes | 3 | - | 36 | - | 0.134 | South-Central Mexico | [3] |
| *Facheiroa squamosa* | Isozymes | 1 | 92.8 | 93 | 0.401 | 0.401 | Northeastern Brazil | [4] |
| *Lophocereus schottii* | Isozymes | 8 | 44.4 | 34 | 0.145 | 0.126 | Sonoran Desert Region | [5] |
| *Lophocereus schottii* | Isozymes | 21 | 90.3 | 50 | 0.214 | 0.144 | Sonoran Desert Region | [1] |
| *Lophocereus schottii* | Isozymes | 21 | 90.6 | - | 0.226 | - | Sonoran Desert Region | [6] |
| *Lophocereus gatesii* | Isozymes | 1 | - | 66 | - | 0.153 | Sonoran Desert Region | [6] |
| *Pachycereus pringlei** | Isozymes | 19 | 91.7 | 62 | 0.212 | 0.200 | Sonoran Desert Region | [1] |
| *Pilosocereus aureispinus* | Isozymes | 1 | 70.5 | 71 | 0.284 | 0.284 | Northeastern Brazil | [4] |
| *Pilosocereus lanuginosus** | Isozymes | 10 | 91.3 | 76 | 0.274 | 0.253 | Northwestern Venezuela | [2] |
| *Pilosocereus machrisii* | Isozymes | 6 | 78.9 | 82 | 0.380 | 0.373 | Eastern Brazil | [4] |
| *Pilosocereus vilaboensis* | Isozymes | 1 | 70.5 | 70.5 | 0.292 | 0.292 | Eastern Brazil | [4] |
| *Pilosocereus tillianus* | Isozymes | 2 | 100 | 98 | 0.352 | 0.35 | Venezuelan Andes | [7] |
| *Polaskia chichipe* | Isozymes | - | - | - | - | 0.504 | South-Central Mexico | [8] |
| *Polaskia chichipe* | Microsatellites | 2 | - | - | - | 0.631 | South-Central Mexico | [9] |
| *Polaskia chende* | Isozymes | 5 | - | 93.3 | - | 0.481 | South-Central Mexico | [10] |
| *Polaskia chende* | Microsatellites | 5 | - | 100 | - | 0.677 | South-Central Mexico | [10] |
| *Praecereus euchlorus* | Isozymes | 6 | 88.2 | 92 | 0.443 | 0.397 | Eastern Brazil | [4] |
| *Stenocereus eruca^a^* | RAPD | 4 | 76 | 72 | - | 0.277 | Sonoran Desert Region | [11] |
| *Stenocereus griseus* | Isozymes | 15 | 100 | 57 | 0.182 | 0.161 | Northwestern Venezuela | [2] |
| *Stenocereus gummosus* | Isozymes | 12 | - | 75 | - | 0.261 | Sonoran Desert Region | [12] |
| *Stenocereus pruinosus* | Isozymes | 3 | - | 100 | - | 0.580 | South-Central Mexico | [13] |
| *Stenocereus pruinosus* | Microsatellites | 3 | - | - | - | 0.720 | South-Central Mexico | [14] |
| *Stenocereus queretaroensis* | ISSR | 5 | 62.9 | 66.1 | - | 0.296 | South-Central Mexico | [15] |
| *Stenocereus thurberi* | Isozymes | 20 | 83.8 | 62 | 0.201 | 0.169 | Sonoran Desert Region | [1] |
| *Stenocereus thurberi* | ISSR | 8 | 66.7 | 54 | 0.207 | 0.175 | Sonoran Desert Region | This study |
| *Stenocereus stellatus* | Isozymes | 6 | - | 86 | - | 0.253 | South-Central Mexico | [16] |
| *Stenocereus stellatus* | Microsatellites | 5 | - | - | - | 0.55 | South-Central Mexico | [17] |
| *Weberbaurocereus weberbaueri** | Isozymes | 1 | - | - | - | 0.257 | Arequipa, Peru | [18] |

In studies that included wild and managed populations (in situ or ex situ) we only included data from wild populations. *N* = number of populations sampled.*Polyploid species. ^a^This species is not strictly a columnar cactus, it grows prostrate.

**References**

1. Hamrick JL, Nason JD, Fleming TH, Nassar JF. Genetic diversity in columnar cacti. In: Fleming TH, Valiente A, editors. Columnar cacti and their mutualist: evolution, ecology, and conservation. Tucson, Arizona: University of Arizona Press; 2002. pp. 122–133.

2. Nassar JM, Hamrick JL, Fleming TH. Population genetic structure of venezuelan chiropterophilous columnar cacti (Cactaceae). Am J Bot. 2003;90: 1628–1637.

3. Tinoco A, Casas A, Luna R, Oyama K. Population Genetics of Escontria chiotilla in Wild and Silvicultural Managed Populations in the Tehuacán Valley, Central Mexico. Genet Resour Crop Evol. 2005;52: 525–538.

4. Moraes EM, Abreu AG, Andrade SCS, Sene FM, Solferini VN. Population genetic structure of two columnar cacti with a patchy distribution in eastern Brazil. Genetica. 2005;125: 311–23.

5. Parker KC, Hamrick JL. Genetic diversity and clonal structure in a columnar cactus, Lophocereus schottii. Am J Bot. 1992;79: 86–96.

6. Nason JD, Hamrick JL, Fleming TH. Historical vicariance and postglacial colonization effects on the evolution of genetic structure in Lophocereus, a Sonoran Desert columnar cactus. Evolution. 2002;56: 2214–2226.

7. Figueredo CJ, Nassar JM, García-Rivas AE, González-Carcacía JA. Population genetic diversity and structure of Pilosocereus tillianus (Cactaceae, Cereeae), a columnar cactus endemic to the Venezuelan Andes. J Arid Environ. 2010;74: 1392–1398.

8. Casas A, Otero-Arnaiz A, Pérez-Negrón E, Valiente-Banuet A. In situ management and domestication of plants in Mesoamerica. Ann Bot. 2007;100: 1101–15.

9. Otero-Arnaiz A, Casas A, Hamrick JL, Cruse-Sanders J. Genetic variation and evolution of Polaskia chichipe (Cactaceae) under domestication in the Tehuacán Valley, central Mexico. Mol Ecol. 2005;14: 1603–11.

10. Contreras-Negrete G, Ruíz-Durán ME, Cabrera-Toledo D, Casas A, Vargas O, Parra F. Genetic diversity and structure of wild and managed populations of Polaskia chende (Cactaceae) in the Tehuacán-Cuicatlán Valley, Central Mexico: insights from SSR and allozyme markers. Genet Resour Crop Evol. 2015;62: 85–101.

11. Clark-Tapia R, Alfonso-Corrado C, Eguiarte LE, Molina-Freaner F. Clonal diversity and distribution in Stenocereus eruca (Cactaceae), a narrow endemic cactus of the Sonoran Desert. Am J Bot. 2005;92: 272–278.

12. Clark-Tapia R, Molina-Freaner F. The genetic structure of a columnar cactus with a disjunct distribution: Stenocereus gummosus in the Sonoran desert. Heredity. 2003;90: 443–50.

13. Parra F, Pérez-Nasser N, Lira R, Pérez-Salicrup D, Casas A. Population genetics and process of domestication of Stenocereus pruinosus (Cactaceae) in the Tehuacán Valley, México. J Arid Environ. 2008;72: 1997–2010.

14. Parra F, Casas A, Peñaloza-Ramírez JM, Cortés-Palomec AC, Rocha-Ramírez V, González-Rodríguez A. Evolution under domestication: ongoing artificial selection and divergence of wild and managed Stenocereus pruinosus (Cactaceae) populations in the Tehuacan Valley, Mexico. Ann Bot. 2010;106: 483–96.

15. Ruán-Tejeda I, Santerre A, Huerta-Martínez FM, Iñiguez-Dávalos LI, Castro-Félix P. Genetic diversity and relationships among wild and cultivated Stenocereus queretaroensis populations in western Mexico. Biochem Syst Ecol. 2014;55: 125–130.

16. Casas A, Cruse-Sanders J, Morales E, Otero-Arnaiz A, Valiente-Banuet A. Maintenance of Phenotypic and Genotypic Diversity in Managed Populations of Stenocereus Stellatus (Cactaceae) by Indigenous Peoples in Central Mexico. Biodivers Conserv. 2006;15: 879–898.

17. Cruse-Sanders JM, Parker KC, Friar EA, Huang DI, Mashayekhi S, Prince LM, et al. Managing diversity: Domestication and gene flow in Stenocereus stellatus Riccob. (Cactaceae) in Mexico. Ecol Evol. 2013;3: 1340–1355.

18. Sahley CT. Bat and Hummingbird Pollination of an Autotetraploid Columnar Cactus, Weberbauerocereus weberbaueri (Cactaceae). Am J Bot. 1996;83: 1329–1336.
